# Association Between Environmental Smoke Exposure in Early Life and ADHD-like Behaviors in Chinese Preschoolers: Findings from Population Survey in Shenzhen

**DOI:** 10.3390/toxics13070534

**Published:** 2025-06-26

**Authors:** Yu-Liang Zhang, Wei-Kang Yang, Esben Strodl, Mao-Lin Zhang, Wei-Qing Chen

**Affiliations:** 1 Department of Epidemiology, School of Public Health, Sun Yat-Sen University, Shenzhen 518107, China; zhangyliang@mail2.sysu.edu.cn; 2 Shenzhen Longhua Maternity and Child Healthcare Hospital, Shenzhen 518000, China; yangweikang@lhfywork.com; 3 School of Psychology and Counselling, Queensland University of Technology, Brisbane, QLD 4000, Australia; e.strodl@qut.edu.au; 4 School of Health Management, Xinhua College of Guangzhou, Guangzhou 510520, China; zhangmlin@mail2.sysu.edu.cn

**Keywords:** environmental tobacco smoke, ADHD, preschoolers, early-life exposure, sensitivity period, gender differences

## Abstract

Environmental tobacco smoke (ETS) exposure is a public health concern linked to neurodevelopmental disorders like attention deficit hyperactivity disorder (ADHD). Prior studies link ETS to ADHD, but gaps remain regarding gender differences, critical exposure windows, and dose–response relationships. This study assessed ETS exposure’s association with ADHD-like behaviors in Chinese preschoolers, evaluating overall risk, critical periods, dose–response relationships, and gender differences. Analyzing data from 64,472 preschoolers, ETS exposure (prenatal; infancy, 0–1; and early childhood, 1–3 years) was assessed via parent questionnaires, and ADHD-like behaviors were measured using the Conners’ Parent Rating Scale-Revised, with associations examined via logistic regression. ETS-exposed children had a 49% higher ADHD-like behavior risk (AOR = 1.49, 95% CI: 1.38–1.62, *p* < 0.001), with dose–response effects: The risk increased from AOR = 1.25 (95% CI: 1.10–1.40) at low exposure to 2.24 (95% CI: 1.63–3.01) at high exposure. Prenatal (AOR = 1.42, 95% CI: 1.17–1.71) and infancy exposures (AOR = 1.43, 95% CI: 1.05–1.90) showed the strongest associations, while early childhood exposure (1–3 years) was non-significant (AOR = 1.04, 95% CI: 0.82–1.29). No gender-specific differences were observed. Early-life ETS exposure, particularly prenatally and in infancy, elevates ADHD-like behavior risk in preschoolers, demonstrating dose–response trends without gender disparity, highlighting the need for universal strategies to reduce such exposures.

## 1. Introduction

Attention deficit hyperactivity disorder (ADHD), a complex neurodevelopmental disorder characterized by persistent inattention and/or hyperactivity-impulsivity, significantly interferes with the function and development of the nervous system [1]. In China, approximately 6.3% of children and adolescents are affected by ADHD, posing substantial societal and economic challenges [2,3]. The disorder often persists into adulthood, where lingering symptoms continue to impair occupational performance, interpersonal relationships, and overall quality of life [4,5,6].

It is well-established that males exhibit a higher prevalence of ADHD and are more likely to receive a diagnosis compared to females [5,6,7,8,9,10]. In 2019, the prevalence was 1611.6 per 100,000 in males, compared to 631.0 per 100,000 in females [4]. This disparity partly stems from overt hyperactive behaviors in boys, which are more easily diagnosed [2,5,8,9,11]. Conversely, girls often show subtler inattention and internalizing behaviors, such as anxiety, leading to underdiagnosis [12,13]. Furthermore, males with ADHD are more likely to present with comorbid conditions, such as substance use disorders and antisocial personality traits, complicating treatment and long-term management [14,15].

The Developmental Origins of Health and Disease (DOHaD) hypothesis emphasizes the critical role of environmental exposures during sensitive early-life periods, such as prenatal and early postnatal stages, in shaping lifelong health trajectories [16,17,18]. These periods of rapid neurodevelopmental change increase vulnerability to external factors [19,20]. Adverse exposures—such as nutritional deficiencies [21], environmental toxins [22], or psychosocial stress [23]—can trigger lasting effects, including epigenetic modifications [24], disruptions in neuronal plasticity [17], and altered organ development [25], increasing risks for conditions like obesity [26], diabetes [27], and neurodevelopmental disorders [28,29,30,31]. The same exposure can yield varied outcomes depending on timing. For example, pneumonia risk varies by living environment and timing [32] while PM2.5 exposure from birth to age two correlates more strongly with childhood ADHD than prenatal exposure [33]. Similar observations have been reported for tobacco smoke and behavioral problems [1], PM10 and rhinitis [34] and benzene and lung function [35]. These findings highlight the need to pinpoint specific environmental factors and their critical windows to inform targeted ADHD interventions.

Environmental tobacco smoke (ETS), commonly referred to as secondhand smoke, contains over 7000 chemicals, including harmful substances such as nicotine and tobacco-specific nitrosamines (TSNAs) [36]. ETS exposure, both prenatal and postnatal, is associated with preterm birth, fetal growth restriction, and neurodevelopmental disorders [37,38,39,40]. During childhood, ETS exposure exacerbates respiratory conditions, impairs cardiometabolic health, negatively impacts cognitive development, and increases the risk of ADHD [3,41,42,43]. Moreover, ETS exposure is associated with long-term cognitive and behavioral consequences, including deficits in cognitive function and language development, mental health issues, and increased behavioral problems [30,36,44,45]. Research also show that ETS exposure during pregnancy [28,41,46] and early childhood (before 6 years old) [28,39,47] and throughout the early years (from 6 to 18 years old) [3,29,41] is associated with an increased risk of developing ADHD. However, whether these exposures have differential effects based on dosage, timing, or a child’s gender remains incompletely understood.

Therefore, this study aimed to explore relationship between ETS exposure and ADHD-like behaviors in preschool children, with a focus on the critical developmental periods, dose–response associations, and gender-specific differences.

## 2. Materials and Methods

### 2.1. Study Population

This study included 67,861 children aged 2 to 7 years and their mothers, recruited from 171 kindergartens in Shenzhen, China through a cross-sectional survey. Eligible participants were children enrolling in these kindergartens for the first time, surveyed once without follow-up. Exclusion criteria included children with severe physical illnesses or mental disorders. Survey methodology details are available in prior studies [48,49].

### 2.2. Data Collection

Data were collected via online structured questionnaires, completed by mothers with guidance from trained kindergarten teachers. These questionnaires captured socio-demographic information about the children and parents, including age, gender, marital status, only-child status, parental education level, family income, reproductive history (maternal and paternal age before pregnancy), sleeping arrangements, prenatal and postnatal ETS exposure, and ADHD diagnoses. To specifically isolate the effects of passive smoke exposure, the study exclusively enrolled mothers with no personal history of active smoking (i.e., never-smokers). In this study, ‘gender’ was defined as biological gender (male/female) based on parental reports at birth [50]. Data on gender identity (e.g., social roles) or intersex/DSD status were not collected.

### 2.3. Data Processing and Exclusion Criteria

To address potential information bias and enhance the representativeness and reliability of the dataset, the following exclusion criteria were applied: (1) mothers younger than 15 years and fathers younger than 18 years; (2) children younger than 3 years (1095 days) or older than 6 years (2200 days); (3) mothers shorter than 1.2 m or weighing less than 35 kg. After exclusions, the final dataset consisted of 64,472 children, ensuring high-quality data for analysis.

### 2.4. Assessment of ETS Exposure

We used a binary variable to assess ETS exposure during early life (1 = exposed, 0 = unexposed), covering pregnancy, birth to 1 year, and 1 to 3 years. Exposure was categorized as “never”, “exposed during pregnancy”, “unexposed until 0 to 1 year old”, or “unexposed until 1 to 3 years old”.

We incorporated two additional questions assessing the quantity and duration of exposure to explore the dose–response relationship. (1) Quantity of Smoke: “The average daily number of cigarettes smoked by household members per day” (coded as: ‘0’ for Never, ‘1’ for 1–5 cigarettes, ‘2’ for 6–10 cigarettes, ‘3’ for 11–15 cigarettes, ‘4’ for 16–20 cigarettes, and ‘5’ for more than 20 cigarettes). (2) Duration of Exposure: “The average daily duration the child was exposed to secondhand smoke per day” (coded as: ‘0’ for Never, ‘1’ for 1–15 min, ‘2’ for 16–30 min, ‘3’ for 31–45 min, ‘4’ for 46–60 min, ‘5’ for 61–90 min, ‘6’ for 91–120 min, and ‘7’ for more than 2 h). Based on the responses to these six quantitative questions (three for exposure time and three for the number of cigarettes), we established two additional variables [51]. (1) Total Score of ETS exposure Level of Cigarettes Number (TSEL-N): sum of scores from the three questions regarding the number of cigarettes smoked daily by family members, with possible values ranging from 0 to 15. (2) Total Score of ETS exposure Level of Time (TSEL-T): sum of scores from the three ETS exposure time questions, with possible values ranging from 0 to 21.

We eventually classified the exposure dose into six levels ranging from 0 to 5, reflecting doses from the lowest to the highest: TSEL-N was divided into 0, 1–2, 3–6, and 7+, with thresholds of 2 and 6 approximating the 25th and 75th percentiles of the exposed population (*n* = 24,419; p25 = 2, p75 = 6). TSEL-T was categorized as 0, 1–3, 4–8, and 9+. Due to the skewness of TSEL-T (p75 = 3 among exposed individuals), higher thresholds (4–8 and 9+) were selected to distinguish moderate-to-high exposure levels. Exact classification standards are presented in Supplementary Materials.

### 2.5. Assessment of ADHD-like Behaviors

ADHD-like behaviors were assessed using the Conners’ Parent Rating Scale-Revised (CPRS-48) [52]. This instrument, translated into Chinese, has shown satisfactory reliability and validity in Chinese populations [53]. ADHD symptoms were measured using the hyperactivity–impulsivity (HI) subscale, consisting of 10 items rated on a scale from 0 to 3 (0 = never, 1 = sometimes, 2 = often, and 3 = frequently). The average score was used both as a continuous variable (range: 0–3) and as a categorical variable, with a threshold of 1.5 identifying significant ADHD symptoms. Higher scores indicated greater levels of hyperactivity.

### 2.6. Confounding Variables

Confounding variables were selected based on the literature [3,39,41,42,47] and analyzed using directed acyclic graphs (DAGs). These variables included maternal and paternal age at pregnancy, family income level, nighttime caregiver during three developmental periods (1~3 months, 4~6 months, and 7~12 months), whether the child was an only child, child age, and maternal and paternal education levels. Variables such as marital status, preterm birth, maternal pre-pregnancy BMI, and abnormal birth weight were excluded to prevent reverse causality bias, as these factors could be influenced by ETS exposure. A detailed DAG diagram is available in the Supplementary Materials.

### 2.7. Statistical Analyses

We conducted a comprehensive analysis to investigate the association between ETS exposure and ADHD-like behaviors in three steps.

In the first step, we employed two binary logistic regression models to examine the association between ETS exposure, evaluated overall and by initial exposure period (pregnancy, 0–1 year, and 1–3 years) and ADHD-like behaviors. The Initial Model included only ETS exposure variables. The Adjusted Model incorporated confounding variables, including maternal and paternal age at pregnancy, family income level, nighttime caregiver during three distinct developmental periods (1 to 3 months old, 4 to 6 months old, and 7 to 12 months old), whether the child was an only child, child age, and maternal and paternal education levels.

In the second step, maintaining the same Initial Model and Adjusted Model, we investigated the dose–response relationship between ETS exposure and ADHD-like behaviors. ETS exposure doses were ranked ordinally, with dose rank “0” as the reference category.

In the third step, maintaining the same Initial Model and Adjusted Model, we extended the analysis by performing a period-specific subgroup examination to evaluate the effects of ETS exposure across three critical time windows: pregnancy, from birth to 1 year old, and from 1 to 3 years old. Participants were categorized by exposure status during these periods, with the “unexposed” subgroup as the reference.

In the final step, maintaining the same Initial Model and Adjusted Model, we conducted a gender-specific analysis to assess gender differences in the association between ETS exposure and ADHD-like behaviors, focusing on overall exposure and initial exposure periods as presented in the main text. To test for gender as a potential moderator, we applied a Z-test to compare the log odds ratios (ln(OR)) between males and females for each exposure category. Additional gender-specific analyses for dose–response and period-specific combination effects are provided in the Supplementary Materials.

Statistical analyses were conducted using R software (version 4.3.2). The findings are reported as crude odds ratios (CORs) and adjusted odds ratios (AORs) with 95% confidence intervals (CIs). We conducted two-tailed tests for all relevant analyses, considering a *p* value of less than 0.05 as the threshold for statistical significance. Additionally, a sensitivity analysis using nested models was conducted to assess the robustness of our findings regarding key subgroup associations.

### 2.8. The Use of Generative AI and AI-Assisted Technologies in the Writing Process

During the preparation of this work, the author(s) used ChatGPT (ChatGPT 4o, o3 and o3-mini), Grok (Grok 3 only), Gemini (Gemini 2.5 pro only), and DeepSeek (Deepseek R1 only) to refine the manuscript by improving language clarity and readability. After using these tools, the author(s) reviewed and edited the content as needed and take full responsibility for the content of the publication.

## 3. Results

### 3.1. Socioeconomic and Early-Life Differences by ETS Exposure in Preschoolers

Significant socioeconomic and caregiving differences were identified between preschoolers exposed to ETS and those unexposed. The ETS-exposed group tended to have parents with lower education levels and younger ages at the time of pregnancy. Families in this group were also more likely to have lower monthly incomes compared to the unexposed group. Males were slightly more represented in the ETS-exposed group, and these children were, on average, older than their unexposed counterparts. Caregiving environments also differed between the two groups. ETS-exposed children were more likely to sleep with grandparents during the first year of life, particularly between 7 and 12 months. These findings are detailed in Table 1.

### 3.2. Overall Impact of ETS Exposure on ADHD-like Behaviors

Exposure to ETS during early life was significantly associated with an increased risk of ADHD-like behaviors (COR = 1.77, 95% CI: 1.64–1.91, *p* < 0.001). After adjusting for potential confounders, the risk remained elevated but was attenuated (AOR = 1.49, 95% CI: 1.38–1.62, *p* < 0.001). Initial period-specific analysis revealed that the initial period of ETS exposure is critical: Exposure during pregnancy was associated with the highest risk of ADHD-like behaviors (AOR = 1.55, 95% CI: 1.42–1.68, *p* < 0.001) while exposure from birth to one year of age also showed a significant association (AOR = 1.49, 95% CI: 1.28–1.73, *p* < 0.001). In contrast, exposure occurred during one to three years old did not demonstrate a statistically significant increase in risk (AOR = 1.04, 95% CI: 0.82–1.29, *p* > 0.05). These findings are detailed in Table 2.

### 3.3. Dose–Dependent Effects of ETS Exposure

A clear dose–response relationship was observed, with higher levels of ETS exposure corresponding to progressively increased risks of ADHD-like behaviors. Children with the lowest exposure level (dose rank 1) had a moderately elevated risk (AOR = 1.25, 95% CI: 1.10–1.40, *p* < 0.001) compared to unexposed children. This risk increased steadily across higher exposure levels, reaching the highest at dose rank 5, where the odds of ADHD-like behaviors were more than double those of the unexposed group (AOR = 2.24, 95% CI: 1.63–3.01, *p* < 0.001). These findings are detailed in Table 3.

### 3.4. Period-Specific Impact of ETS Exposure on ADHD-like Behaviors

The risk of ADHD-like behaviors varied by the timing of ETS exposure: For children exposed during a single period, exposure during pregnancy or the first year of life was associated with significantly higher risk (pregnancy: AOR = 1.42, 95% CI: 1.17–1.71, *p* < 0.001; 0–1 year: AOR = 1.43, 95% CI: 1.05–1.90, *p* < 0.05), while exposure during 1–3 years showed no significant effect (AOR = 1.04, 95% CI: 0.82–1.29, *p* > 0.05). For children exposed during two periods, the risk was higher, particularly for exposure during both 0–1 year and 1–3 years (OR = 1.51, 95% CI: 1.27–1.78, *p* < 0.001). For children exposed during all three periods, the highest risk was observed, nearly doubling the likelihood of ADHD-like behaviors (OR = 1.60, 95% CI: 1.46–1.76, *p* < 0.001). These findings are detailed in Table 4.

### 3.5. Gender-Specific Impact of ETS Exposure on ADHD-like Behaviors

ETS exposure in early life was significantly associated with an increased risk of ADHD-like behaviors in both males (AOR = 1.55, 95% CI = 1.40~1.71, *p* < 0.001) and females (AOR = 1.38, 95% CI = 1.21~1.58, *p* < 0.001). However, despite males and females exhibiting differences across each group, the results of statistical tests suggested that there was not enough certainty to consider a significant difference between the two. These findings are detailed in Table 5. 

We also conducted a gender-specific dose–response analysis and a gender-specific period-specific analysis. Detailed information can be viewed in Supplementary Materials.

## 4. Discussion

This study is the first study to comprehensively evaluate the impact of environmental tobacco smoke (ETS) exposure on ADHD-like behaviors in Chinese preschool children using large population survey, while also exploring gender-specific differences, timing, and dose–response relationships. Our findings demonstrate a robust association between ETS exposure and increased ADHD risk, with higher exposure levels corresponding to progressively greater risks. The timing of exposure emerged as critical, with prenatal and early postnatal periods showing the strongest associations. However, the impact of ETS on ADHD did not exhibit significant gender-specific differences.

Previous studies have revealed important insights into the relationship between ETS exposure and ADHD. Initially, several studies have consistently demonstrated a significant association between ETS exposure and an increased risk of developing ADHD in children [28,39,41,42,54]. Simultaneously, research indicates that ETS exposure not only elevates the overall risk of ADHD but also exerts differential effects on its subtypes; that is, prenatal ETS exposure has been linked to a heightened risk for the inattentive subtype (ADHD-I), while postnatal ETS exposure is more strongly associated with the hyperactive–impulsive subtype (ADHD-HI) [38]. Maternal cotinine levels during the third trimester have been associated with hyperactivity and inattention rather than conduct problems in children [3]. Prenatal exposure has also been implicated in internalizing problems [30]. In addition, the influence of ETS exposure on ADHD is not uniform; the effects vary according to multiple factors such as the timing [47,55] and dosage [3,54] as well as factors relating to the specific population such as mode of exposure (maternal active smoking [39] or passive smoking [29,42]). Our findings not only concur with previous evidence linking ETS exposure and elevated ADHD risk, but they also extend our understanding by showing how the dose and time window of exposure further shape this relationship.

Beyond these known impacts, previous studies [3,28,54] have demonstrated that the health effects of ETS increase with increasing exposure dose. Our findings are consistent with this finding, with strong support for the existence of a dose–response relationship in the effects of ETS on ADHD. However, although the dose–response relationship between ETS exposure and ADHD risk is evident, with higher exposure levels consistently associated with increased risk [56], the specific details underlying this association remain elusive. Previous studies investigating ETS exposure, across various health outcomes, have commonly employed indirect measures such as questionnaires assessing exposure duration and household smoking frequency [28,54,57,58,59], or direct biological markers such as blood cotinine levels [30,41,43] to estimate ETS exposure doses, yet these approaches face limitations. Questionnaire-based assessments struggle to accurately quantify exposure levels, while cotinine measurements, though precise, fail to reflect long-term exposure over time. These methodological limitations impede accurate quantification of the dose–response relationship. A promising direction for future research is, therefore, the integration of these two approaches. For instance, a hybrid design combining broad questionnaire data with periodic biochemical validation in a subsample could offer a more robust assessment. Although such a design may be more costly and limit sample size, it would provide higher-quality data crucial for a more profound understanding of the neurodevelopmental impact of ETS exposure.

To better understand how ETS influences ADHD risk in the context of these dose-response patterns, it is essential to explore the underlying biological mechanisms through which ETS exerts its effects. The mechanisms by which ETS increases ADHD risk remain unclear and ETS may exert its effects through complex pathways. Direct effects include structural and functional alterations in the central nervous system [60,61], while indirect mechanisms, such as reduced breastfeeding, may also contribute [62]. These mechanisms likely operate in combination. For instance, Wang et al. [63] reported significant gene–environment interactions between ETS and the ADRA2A rs553668 gene in relation to ADHD and ADHD-ODD. Similarly, Miyake et al. [64] suggested that the association between maternal smoking during pregnancy and ADHD symptoms at school age may be mediated by DNA methylation of the GFI1 gene. These mechanisms are particularly relevant during early developmental stages, where heightened vulnerability to ETS exposure may amplify its neurodevelopmental impact.

Our study also highlights the critical importance of timing in ETS exposure, identifying the prenatal and early postnatal period from birth to one year old as particularly vulnerable stages for ADHD risk. This aligns with findings by Lin et al. [28], who reported a stronger risk of ADHD among children exposed to ETS during pregnancy compared to other periods. Similarly, a meta-analysis by Dong et al. [39] revealed that mothers who quitted smoking before pregnancy had a significantly reduced risk of having children with ADHD compared to those who quit during the first trimester. Reinforcing this, a systematic review that included 20 studies [55] reported an AOR of 1.60 (95% CI: 1.45–1.76) for ADHD risk in offspring associated with prenatal smoking, closely aligning with our prenatal exposure findings. This sensitivity to early-life exposures likely stems from developmental processes, including metabolic programming influenced by mitochondrial dysfunction, epigenetic modifications, and disrupted glucose metabolism [21], as well as heightened oxidative stress sensitivity and environmental vulnerability during critical stages of organ development [65]. These mechanisms are implicated in various conditions, including obesity [26,66,67], airway diseases [68], small-for-gestational-age [69,70], behavioral problems [29,54], and cardiometabolic risk [43]. In addition, this early-life vulnerability extends to various exposures, with studies demonstrating stronger effects from polycyclic aromatic hydrocarbons [71], maternal stress [23], and nutritional intake [21] during these stages compared to later development. Our findings strongly support early life as a sensitive window during which ETS exposure may predispose individuals to ADHD. In addition, the complexity of these period-specific interactions is further underscored by our sensitivity analysis (detailed information can be viewed in Supplementary Materials). It revealed that the association for certain subgroups, particularly for combined prenatal and infant exposure, was strongly influenced by socio-demographic factors, highlighting their critical role in shaping a child’s vulnerability profile during these early years.

It is noteworthy that in our study, ETS exposure during 1 to 3 years old was not significantly associated with ADHD-like behaviors. However, as substantial evidence indicates that ETS exposure in early childhood is a significant risk factor for various adverse health outcomes [1,29,32,35,54], this null finding should be interpreted with caution and does not necessarily imply a “safe” period of exposure. One possibility is that while the prenatal and infancy periods are critical windows for neurodevelopmental insults, the toddler years may be a more sensitive period for other adverse health outcomes, such as respiratory illnesses [32,68] or cardiovascular health [43]. Alternatively, it is plausible that a lagged effect [72,73,74] exists, where the neurodevelopmental impact of ETS exposure during this period only manifests as detectable behavioral problems later in childhood. Future longitudinal studies are needed to disentangle these distinct possibilities.

Beyond the dose–response relationship and sensitive periods, we examined potential gender differences in the impact of ETS exposure on ADHD-like behaviors. Consistent with prior research [5,29,54,75,76], our findings confirm that ETS exposure is associated with increased ADHD risk in children of both genders. However, unlike some studies [1,75,76] suggesting greater male susceptibility, our analysis revealed no statistically significant gender differences in this association, either overall or across specific exposure periods (*p* > 0.05, Table 5). Notably, the robust associations observed during pregnancy (male: AOR = 1.63, *p* < 0.001; female: AOR = 1.40, *p* < 0.001) and birth to 1 year (male: AOR = 1.47, *p* < 0.001; female: AOR = 1.54, *p* < 0.001) in both genders reinforce the concept of an early vulnerable stage [37,77,78,79]. This finding is particularly noteworthy. While ADHD prevalence is higher in males [5,6,8], our results suggest that the biological vulnerability to the neurotoxic effects of ETS may not differ significantly by gender. This implies that the higher prevalence of ADHD observed in boys is likely driven by other factors, such as differences in symptom expression [12,13] or diagnostic practices [2,5,8], rather than a greater inherent susceptibility to this specific environmental risk factor.

Based upon the large survey size, this study contributes to the understanding of critical periods in the impact of ETS exposure on ADHD. Despite the strengths of our study, there are several limitations to consider. First, while the overall sample size was large, some subgroup analyses, particularly at higher dose–response levels, were based on smaller numbers of participants, which may have limited the statistical power to detect more subtle associations. Second, the use of a rating scale to identify ADHD-like behaviors, while validated, serves as a screening tool rather than a formal clinical diagnosis and may not capture the full clinical complexity of the disorder. Third, the cross-sectional design of this study inherently limits our ability to establish a definitive causal relationship. Unlike longitudinal cohort studies, which can track developmental trajectories over time, our one-time data collection cannot fully determine the temporal sequence between ETS exposure and the onset of ADHD-like behaviors. Finally, our assessment of ETS exposure relied on parental self-reports, which may be subject to recall bias or social desirability bias, potentially leading to some degree of exposure misclassification. Therefore, future prospective cohort studies using formal clinical assessments for ADHD are warranted to overcome these limitations and corroborate our findings.

Our study demonstrates that ETS exposure in early life elevates ADHD-like behavior risk in children through a dose–response relationship, with pregnancy and the first year of life as critical vulnerability windows, affecting both genders similarly. These findings highlight the public health imperative to minimize ETS exposure, particularly during these sensitive periods, through targeted interventions like smoking cessation programs for expectant and new parents. By showing consistent ETS effects across genders, this research supports universal prevention strategies to safeguard neurodevelopmental health. Future studies should investigate factors driving these effects, potentially beyond gender-specific influences, such as universal developmental or environmental contributors, using longitudinal designs and improved exposure assessments to refine prevention strategies.

## 5. Conclusions

This study revealed that early-life environmental tobacco smoke exposure significantly elevates the risk of ADHD-like behaviors among Chinese preschoolers, an effect characterized by a clear dose–response relationship. Crucially, the prenatal period and infancy were identified as critical windows of heightened neurodevelopmental vulnerability to these detrimental exposures. Crucially, while ADHD is diagnosed more frequently in males, our findings suggest a comparable biological susceptibility to the neurotoxic effects of ETS across genders. Collectively, these findings underscore the profound risks of ETS to early brain development and provide a scientific basis for universal prevention strategies that target all children, regardless of gender. Such measures should prioritize the protection of expectant mothers and infants from ETS to effectively mitigate the burden of ADHD-like behaviors and foster healthier neurodevelopmental outcomes for all children.

## Figures and Tables

**Table 1:** Baseline characteristics of preschoolers by environmental tobacco smoke (ETS) exposure status ^1^.

Characteristics	Unexposed to ETS (*n* = 40,053)	Exposed to ETS (*n* = 24,419)	*p*-Value ^2^
Gender (%)			0.002
Male	21,510 (53.7%)	13,419 (55.0%)	
Female	18,543 (46.3%)	11,000 (45.0%)	
Child age (mean (SD), Days)			<0.001
	1673.77	1691.77	
Maternal education level (%)			<0.001
Junior high school or below	8592 (21.5%)	7886 (32.3%)	
High school or secondary vocation school	11,184 (27.9%)	7995 (32.7%)	
Three-year college	10,285 (25.7%)	5311 (21.7%)	
Bachelor degree or above	9992 (24.9%)	3227 (13.2%)	
Paternal education level (%)			<0.001
Junior high school or below	6321 (15.8%)	7011 (28.7%)	
High school or secondary vocation school	9900 (24.7%)	7763 (31.8%)	
Three-year college	9729 (24.3%)	5180 (21.2%)	
Bachelor degree or above	14,103 (35.2%)	4465 (18.3%)	
Family monthly income (Ұ, %)			<0.001
less than 5 k	5296 (13.2%)	4425 (18.1%)	
5 k to 10 k	9810 (24.5%)	7657 (31.4%)	
10 k to 20 k	13,752 (34.3%)	7805 (32%)	
more than 20 k	11,195 (28%)	4532 (18.6%)	
Maternal age before pregnancy (mean (SD))			<0.001
	27.39	26.04	
Paternal age before pregnancy (mean (SD))			<0.001
	30.08	28.78	
Whether an only child (%)			<0.001
No	20,794 (51.9%)	14,096 (57.7%)	
Yes	19,259 (48.1%)	10,323 (42.3%)	
Sleeping partner during 0~3 months (%)			<0.001
sleep alone	624 (1.6%)	325 (1.3%)	
sleep with relative or nanny	312 (0.8%)	182 (0.7%)	
sleep with grandparents	2382 (5.9%)	1660 (6.8%)	
sleep with parents	36,735 (91.7%)	22,252 (91.1%)	
Sleeping partner during 4~6 months (%)			<0.001
sleep alone	542 (1.4%)	274 (1.1%)	
sleep with relative or nanny	336 (0.8%)	198 (0.8%)	
sleep with grandparents	3389 (8.5%)	2528 (10.4%)	
sleep with parents	35,786 (89.3%)	21,419 (87.7%)	
Sleeping partner during 7~12 months (%)			<0.001
sleep alone	445 (1.1%)	210 (0.9%)	
sleep with relative or nanny	425 (1.1%)	265 (1.1%)	
sleep with grandparents	6095 (15.2%)	4673 (19.1%)	
sleep with parents	33,088 (82.6%)	19,271 (78.9%)	

^1^ Note: Values represent means (SD) for continuous variables and *n* (%) for categorical variables. ^2^ *p*-Value: derived from chi-square tests for categorical variables and *t*-tests for continuous variables.

**Table 2:** Association between ETS exposure in early life and ADHD-like behaviors.

Children ETS Exposure	Total (N)	Cases (N, %)	COR (95% CI) ^1^	AOR (95% CI) ^2^
ETS exposure				
No	40,053	1338 (3.3%)	1.00	1.00
Yes	24,419	1407 (5.8%)	1.77 (1.64–1.91) ***	1.49 (1.38–1.62) ***
Initial period of ETS exposure				
Never	38,715	1338 (3.3%)	1.00	1.00
Pregnancy	17,263	1107 (6.0%)	1.86 (1.71–2.01) ***	1.55 (1.42–1.68) ***
From birth to one year old	3694	217 (5.5%)	1.70 (1.46–1.97) ***	1.49 (1.28–1.73) ***
From one to three years old	2055	83 (3.9%)	1.17 (0.93–1.46)	1.04 (0.82–1.29)

^1^ COR: crude odds ratio; ^2^ AOR: adjusted odds ratio, with adjustment for child’s age, parental educational level, parental age at the time of the child’s birth, family income, and marriage status. ***: *p* < 0.001.

**Table 3:** Dose–response analysis of ETS exposure on ADHD-like behaviors.

ETS Exposure Doses ^1^	Total (N)	Case (N, %)	COR (95% CI) ^2^	AOR (95% CI) ^3^
0	40,053	1338 (3.3%)	1.00	1.00
1	7442	356 (4.8%)	1.45 (1.29–1.64) ***	1.25 (1.10–1.40) ***
2	10,742	604 (5.6%)	1.72 (1.56–1.90) ***	1.43 (1.29–1.58) ***
3	4099	280 (6.8%)	2.12 (1.85–2.42) ***	1.82 (1.59–2.08) ***
4	1551	120 (7.7%)	2.43 (1.99–2.93) ***	2.07 (1.70–2.51) ***
5	585	47 (8.0%)	2.53 (1.84–3.38) ***	2.24 (1.63–3.01) ***

^1^ ETS exposure doses: categorized into six levels (0–5) based on the total score of ETS exposure level of time (TSEL-T) and cigarette number (TSEL-N), with higher levels indicating greater exposure intensity and duration. ^2^ COR: crude odds ratio. ^3^ AOR: adjusted odds ratio, with adjustment for child’s age, parental educational level, parental age at the time of the child’s birth, family income, and marriage status. ***: *p* < 0.001.

**Table 4:** Combination effect of ETS exposure in three periods of early life on ADHD-like behaviors.

ETS Exposure Periods			Total (N)	Case (N, %)	COR (95% CI) ^1^	AOR (95% CI) ^2^
Pregnancy	From Birth to One Year Old	From One to Three Years Old				
No	No	No	40,053	1338 (3.3%)	1.00	1.00
Yes	No	No	2138	83 (3.9%)	1.62 (1.33–1.93) ***	1.42 (1.17–1.71) ***
No	Yes	No	903	48 (5.3%)	1.62 (1.19–2.16) **	1.43 (1.05–1.90) *
No	No	Yes	3008	169 (5.6%)	1.17 (0.93–1.46)	1.04 (0.82–1.29)
Yes	Yes	No	2345	124 (5.3%)	1.43 (1.05–1.90) *	1.22 (0.90–1.63)
Yes	No	Yes	1257	69 (5.5%)	1.68 (1.30–2.14) ***	1.41 (1.09–1.80) **
No	Yes	Yes	1018	48 (4.7%)	1.72 (1.46–2.02) ***	1.51 (1.27–1.78) ***
Yes	Yes	Yes	13,750	866 (6.3%)	1.95 (1.78–2.12) ***	1.60 (1.46–1.76) ***

^1^ COR: crude odds ratio. ^2^ AOR: adjusted odds ratio, with adjustment for child’s age, parental educational level, parental age at the time of the child’s birth, family income, and marriage status. ***: *p* < 0.001. **: *p* < 0.01. *: *p* < 0.05.

**Table 5:** Gender-specific association between ETS exposure in early life and ADHD-like behaviors.

ETS Exposure	Male			Female			*p*-Value ^2^
	Total (N)	Cases (*n*, %)	AOR (95% CI) ^1^	Total (N)	Cases (*n*, %)	AOR (95% CI) ^1^	
ETS exposure							
No	21,510	848 (4%)	1.00	18,543	490 (3%)	1.00	
Yes	13,419	948 (7%)	1.55 (1.40~1.71) ***	11,000	459 (4%)	1.38 (1.21~1.58) ***	0.18
Initial period of ETS exposure							
Never	21,510	848 (4%)	1.00	18,543	490 (3%)	1.00	
Pregnancy	10,051	751 (7%)	1.63 (1.46~1.81) ***	8319	356 (4%)	1.40 (1.21~1.62) ***	0.10
From birth to one year old	2130	138 (6%)	1.47 (1.21~1.77) ***	1781	79 (4%)	1.54 (1.20~1.96) ***	0.76
From one to three years old	1238	59 (5%)	1.08 (0.82~1.41)	900	24 (3%)	0.89 (0.57~1.33)	0.45

^1^ AOR: adjusted odds ratio, with adjustment for child’s age, parental educational level, parental age at the time of the child’s birth, family income, and marriage status. ^2^ *p*-Value: calculated using a Z-test to compare the odds between males and females. ***: *p* < 0.001.

## Data Availability

The data presented in this study are available on request from the corresponding author. Due to confidentiality and ethical restrictions related to participant privacy, the data are not publicly available.

## Supplementary Materials

The following supporting information can be downloaded at: https://www.mdpi.com/article/10.3390/toxics13070534/s1, Figure S1: DAGs of Confounding Variables; Table S1: Exact classification standards of ETS exposure doses; Table S2: Dose Response analysis of Gender-Specific ETS Exposure on ADHD-like Behaviors; Table S3: Combination effect of ETS Exposure in three period of early life on ADHD-like Behaviors by Gender. Table S4: Sensitivity Analysis of Confounder Adjustment for the Association between Combined Prenatal & Infant ETS Exposure and ADHD-like Behaviors.

## Abbreviations

The following abbreviations are used in this manuscript:

ETS	Environmental Tobacco Smoke
ADHD	Attention Deficit Hyperactivity Disorder
DOHaD	Developmental Origins of Health and Disease
DAGs	Directed Acyclic Graphs
COR	Crude Odds Ratio
AOR	Adjusted Odds Ratio
