# Supplementary material for: Association Between Environmental Smoke Exposure in Early Life and ADHD-like Behaviors in Chinese Preschoolers: Findings from Population Survey in Shenzhen
Source: Toxics. 2025 Jun 26;13(7):534. doi: 10.3390/toxics13070534 (PMC12300800; doi:10.3390/toxics13070534)
Supplement: Supplementary file 1 [file toxics-13-00534-s001.zip › toxics-3689332-supplementary.pdf]

(A)

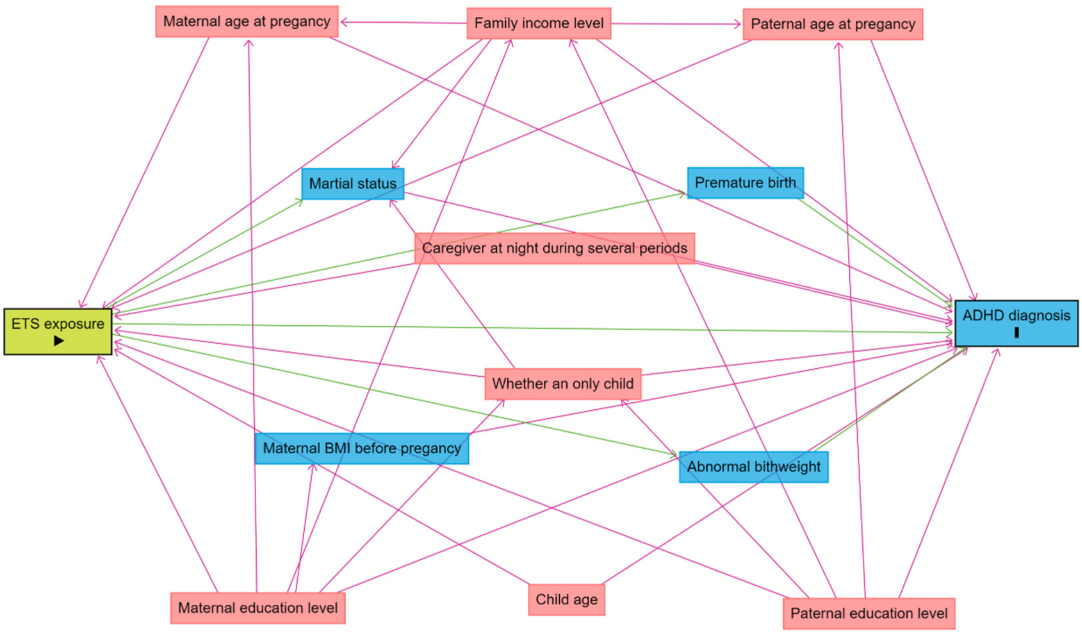

(**B**)

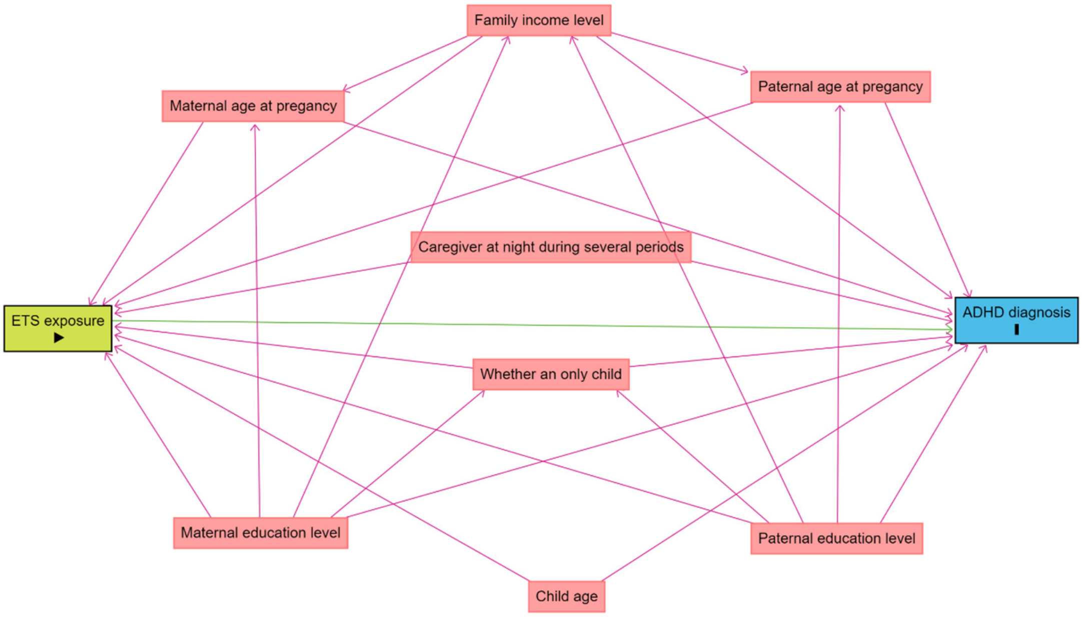

Figure S1. DAGs of Confounding Variables, including (**A**) confounding variables before selection and (**B**) confounding variables after selection.

Table S1. Exact classification standards of ETS exposure doses

Dose of TSEL-N [1]	Dose of TSEL-T [2]			
	**0**	**1~3**	**4~8**	**9~**
0	0	-	-	-
1~2	-	1	2	3
3~6	-	2	3	4
7~	-	3	4	5

[1] TSEL-N: Total Score of ETS exposure Level of Cigarettes Number, the sum of scores from the three questions regarding the number of cigarettes smoked daily by family members, with possible values ranging from 0 to 15. [2] TSEL-T: Total Score of ETS exposure Level of Time, the sum of scores from the three ETS exposure time questions, with possible values ranging from 0 to 21.

Table S2. Dose Response analysis of Gender-Specific ETS Exposure on ADHD-like Behaviors

ETS exposure doses	Male			Female			*p*-Value [2]
	**Total (N)**	**Case (N, %)**	**AOR (95% CI) [1]**	**Total (N)**	**Case (N, %)**	**AOR (95% CI) [1]**	
0	21510	848(0.04)	1.00	18543	490(3%)	1.00	1.00
1	4111	242(0.06)	1.29 (1.11~1.49) ***	3331	114(3%)	1.14 (0.92~1.4)	0.66
2	5884	418(0.07)	1.54 (1.35~1.74) ***	4858	186(4%)	1.23 (1.03~1.47) *	0.36
3	2238	178(0.08)	1.79 (1.50~2.11) ***	1861	102(5%)	1.88 (1.50~2.34) ***	0.06
4	853	78(0.09)	2.07 (1.61~2.63) ***	698	42(6%)	2.06 (1.46~2.83) ***	0.94
5	333	32(0.10)	2.25 (1.52~3.22) ***	252	15(6%)	2.14 (1.20~3.53) **	0.78

[1] AOR: Adjusted odds ratio, with adjustment for child's gender, age, parental educational level, parental age at the time of the child's birth, family income and marriage status. [2] *p*-Value: Calculated using a Z-test to compare the odds between males and females. ***: p<0.001. **: p<0.01. *: p<0.05

Table S3. Combination effect of ETS Exposure in three period of early life on ADHD-like Behaviors by Gender

ETS exposure			Male			Female			*p*-Value [2]
**Preg-nancy**	**From birth to one year old**	**From one to three years old**	**Total (N)**	**Case (N, %)**	**AOR (95% CI) [1]**	**Total (N)**	**Case (N, %)**	**AOR (95% CI) [1]**	
No	No	No	21510	848(3.9%)	1.00	18543	490(2.6%)	1.00	1.00
Yes	No	No	1238	80 (6.4%)	1.45 (1.13~1.83) **	900	44 (4%)	1.37 (0.99~1.86)	0.78
No	Yes	No	499	33 (6.6%)	1.51 (1.03~2.14) *	404	15 (3.7%)	1.25 (0.71~2.04)	0.56
No	No	Yes	1631	59 (4.8%)	1.08 (0.81~1.40)	1377	24 (2.7%)	0.90 (0.58~1.33)	0.47
Yes	Yes	No	1246	30 (5.5%)	1.22 (0.82~1.75)	1099	18 (3.8%)	1.24 (0.74~1.95)	0.96
Yes	No	Yes	687	48 (7%)	1.53 (1.11~2.05) **	570	21 (3.7%)	1.19 (0.73~1.81)	0.36
No	Yes	Yes	543	105(6.4%)	1.45 (1.17~1.78) ***	475	64 (4.6%)	1.60 (1.21~2.08) ***	0.57
Yes	Yes	Yes	7575	593(7.8%)	1.68 (1.50~1.89) ***	6175	273(4.4%)	1.44 (1.23~1.69) ***	0.12

[1] AOR: Adjusted odds ratio, with adjustment for child's gender, age, parental educational level, parental age at the time of the child's birth, family income and marriage status. [2] *p*-Value: Calculated using a Z-test to compare the odds between males and females. ***: p<0.001. **: p<0.01. *: p<0.05

Table S4. Sensitivity Analysis of Confounder Adjustment for the Association between Combined Prenatal & Infant ETS Exposure and ADHD-like Behaviors.

Model / Covariates Adjusted	AOR [1]	*p*-Value [2]	95% CI
Model 1: Adjusted for child's age	1.45	0.014	1.06~1.92
Model 2: Model 1 + Parental education & Family income	1.32	0.068	0.97~1.75
Model 3: Model 1 + Parental age at birth & Marital status	1.31	0.070	0.96~1.75
Model 4: Fully Adjusted Model [3]	1.23	0.166	0.90~1.64

[1] AOR: Adjusted odds ratio, with adjustment for child's age, parental educational level, parental age at the time of the child's birth, family income and marriage status. [2] *p*-Value: Calculated using a Z-test [3] Includes child's age, parental education level, family income, parental age at the time of the child's birth, and marriage status.
